# Pesticide Residues in Canned Foods, Fruits, and Vegetables: The Application of Supercritical Fluid Extraction and Chromatographic Techniques in the Analysis

**DOI:** 10.1100/tsw.2003.85

**Published:** 2003-12-11

**Authors:** Mohamed H. EL-Saeid

**Affiliations:** Department of Food Science and Technology, AL-Azhar University, Nasr City, Cairo, Egypt

**Keywords:** toxicology, pesticides, imported fruits and vegetables, SFE and SFC method

## Abstract

Multiple pesticide residues have been observed in some samples of canned foods, frozen vegetables, and fruit jam, which put the health of the consumers at risk of adverse effects. It is quite apparent that such a state of affairs calls for the need of more accurate, cost-effective, and rapid analytical techniques capable of detecting the minimum concentrations of the multiple pesticide residues. The aims of this paper were first, to determine the effectiveness of the use of Supercritical Fluid Extraction (SFE) and Supercritical Fluid Chromatography (SFC) techniques in the analysis of the levels of pesticide residues in canned foods, vegetables, and fruits; and second, to contribute to the promotion of consumer safety by excluding pesticide residue contamination from markets. Fifteen different types of imported canned and frozen fruits and vegetables samples obtained from the Houston local food markets were investigated. The major types of pesticides tested were pyrethroids, herbicides, fungicides, and carbamates.By using these techniques, the overall data showed 60.82% of the food samples had no detection of any pesticide residues under this investigation. On the other hand, 39.15% different food samples were contaminated by four different pyrethroid residues ± RSD% ranging from 0.03 ± 0.005 to 0.05 ± 0.03 ppm, of which most of the pyrethroid residues were detected in frozen vegetables and strawberry jam. Herbicide residues in test samples ranged from 0.03 ± 0.005 to 0.8 ± 0.01 ppm. Five different fungicides, ranging from 0.05 ± 0.02 to 0.8 ±0.1 ppm, were found in five different frozen vegetable samples. Carbamate residues were not detected in 60% of investigated food samples. It was concluded that SFE and SFC techniques were accurate, reliable, less time consuming, and cost effective in the analysis of imported canned foods, fruits, and vegetables and are recommended for the monitoring of pesticide contaminations.

### INTRODUCTION

In some countries, over 51% of imported foods, fruits, and vegetables samples have been reported with the limit of quantitation set at 0.01 μ/g, but the limit of detection was 0.001 μ/g[1]. For example, the twin-island state of Trinidad and Tobago produces much of the fresh fruit and vegetables consumed locally. Although some are exported to Europe, approximately 1,500 tons (on average) are imported into the U.S. annually, which are contaminated with a wide range of pesticides. Furthermore, a market basket survey of produce conducted between October 1996 and May 1997 in Trinidad for pesticides showed that 10% of the produce exceeded the internationally acceptable maximum residue limits (MRLs) for the respective pesticides[2]. Violations of MRLs have also been observed in imported samples with detectable residues above 0.01 μ/g in 55% of domestic and 38% of imported samples (<10% of the MRL). Of all the samples, 2.4% contained more than five different pesticides; tomatoes, strawberries, apples, and citrus fruits tended to have more multiple residues[1].

In Canada, on the other hand, among 16,198 imported agricultural food products, 464 of 3,193 residue findings exceeded MRLs, corresponding to a violation rate of 2.86%. Of these, 32 (0.55%) were in violation of Canadian MRLs. One-half of domestic violations resulted from commodity-pesticide combinations for which there were no Canadian approvals[3]. In Sweden, published data on pesticide residues in domestically grown fruits and vegetables showed that the proportion of cases of reported residues was higher than 20% of the MRL. Residues in imported food crops of the same type increased from 31% to 37%[4]. To improve the worrisome global situation, it would seem appropriate that the United Nations Food and Agriculture Organization pay greater attention to the need for promotion of restrictions on availability of highly toxic and other pesticides, as recommended by FAO and WHO in 1975 (WHO/FAO 1975)[4].

In 1992–1993, the U.S. Food and Drug Administration (FDA) conducted a statistically based study of pesticide residues in domestic and imported pears and tomatoes. For pears, 710 domestic and 949 imported samples were collected and analyzed, where 79% of the domestic and 72% of the imported samples had detectable residues[5]. The statistically weighted violation rates for domestic and imported tomatoes were 1.9 and 7.0%, respectively[5]. The aim of this paper was to determine the effectiveness of the use of Supercritical Fluid Extraction (SFE) and Supercritical Fluid Chromatography (SFC) techniques in the analysis of the levels of pesticide residues in canned foods, vegetables, and fruits; and to contribute to the promotion of consumer safety by excluding pesticide residue contamination from markets[5,6]. Fifteen different types of canned and frozen fruits and vegetable samples obtained from the Houston local food markets were investigated. The major types of pesticides tested were pyrethroids, herbicides, fungicides, and carbamates.

In many agriculture areas, pesticides are used intensely. In these areas, fruits and vegetables are very important, and therefore acaricides, insecticides, fungicides, and herbicides are applied on the agriculture areas[6,7]. Many different types of pesticides[8,9] are used extensively, leading to the contamination of pesticide residues in fruits and vegetables. Pyrethroid[10], herbicide[11], fungicide[12,13,14], and carbamate[15,16,17,18] residues are frequently detected in fruit and vegetable crops. The present contribution is an overview of the application of the multiresidue methods of extraction by SFE and analysis by SFC in the analysis of pyrethroid, herbicide, fungicide, and carbamate residues[19,20,21,22] in canned foods, fruits, juices, and frozen vegetables.

### AIMS OF THE INVESTIGATION

The aims of this investigation were twofold: (1) to determine the effectiveness of the use of SFE and SFC techniques in the analysis of the levels of pesticide residues in canned foods, vegetables, and fruits collected from Houston local markets and (2) to contribute to the promotion of consumer safety by excluding pesticide residue contamination from markets. Indirectly, the outcome will contribute to the achievement of risk reduction measured mainly in terms of pesticide residues in foods (both imported and grown domestically), and acute health hazard to the users.

### MATERIALS AND METHODS

#### Sample Collection and Preparation

Fifteen different types of fruit juice (guava, mango, and fruit cocktail), frozen vegetable (mixed, molukhia, artichoke, colocassia, aubergines, beans, and okra), canned food (fava beans and red hot peppers), and jam (fig, orange, and strawberry) samples were obtained from local Houston food markets. The samples were each homogenized, then divided into two portions — one as a blank or control sample for Quality Control and Quality Assurance, and the other for spiking with each single pesticide in each group of investigated pesticides. Both samples were subjected to freeze drying. The freeze-dried powdered food samples were stored in 1-kg glass containers at –20ºC until analysis. To calculate relative standard deviation (RSD%), three replicate spikes were analyzed.

#### Recovery Assays

Untreated food samples (15) were spiked with each single pesticide in each group of investigated pesticides. Recovery assays were performed at levels ranging from 0.5–1.0 μg/g. The spiked samples were then homogenized and allowed to equilibrate for 1 h prior to freeze drying. Three replicates were analyzed to calculate the recovery and RSD%.

#### Pesticide Standards

Thirty-five pure pesticide standards commonly used for the agricultural purposes were obtained from Chemservice, Inc. (West Chester, PA) and EPA (Research Triangle Park NC). All obtained investigation pesticide samples consisted of 4 pesticide groups, namely: 8 pyrethroids and their metabolites (allethrin, resmethrin, phenothrin, permethrin, tetramethrin, cypermethrin, deltamethrin, and phenoxy benzyl alcohol), 8 herbicides (trifluralin, tillam, chlorthal, alachlor, propazin, terbuthylazin, atrazin, and simazin), 7 fungicides (PCNP, CDEC, dichlon, captan, captafol, thiram, and carboxin), and 12 carbamates (benthiocarb, pirimicarb, aldicarb, bendiocarb, baygon, BDMC, methiocarb, barban, carbaryl, benomyl, methomyl, and oxamyl).

#### Pesticide Extraction

The pesticide extraction methods of Khan[21] were modified by EL-Saeid[23] and used in the present investigation. An SFE model 7680 T (Hewlett-Packard) was used, which included a solid phase sorbing trap with 30 mm of Hypersil ODS into which the $CO_2$ extraction solvent was decompressed during collection. Dry food samples (5 g) were transferred into the extraction thimble. The extraction process was carried out in three steps; in the first step $CO_2$ density was 0.25 g/ml, at an extraction pressure of 77

bars (1,117 psi), chamber temperature 40°C, and $CO_2$ flow rate 1.0 ml/min. In the second step, $CO_2$ density was 0.67 g/ml, set at an extraction pressure of 239 bars (3,469 psi). The chamber temperature was 80°C, and $CO_2$ flow rate was 2.5 ml/min. The nozzle temperature was set at 45°C in both steps. The sample extract was collected on the ODS sorbing trap at 10°C. The extracted sample was eluted from the trap with 1.5 ml of methanol at a flow rate of 0.4 ml/min and a trap temperature of 40 EC and collected in auto sampler vials placed in a fraction collector. The ODS trap was regenerated between extractions by rinsing with 2 ml of methylene chloride followed by 2 ml of methanol at 1 ml/min to the waste. The same conditions were applied in the third step as to the second step except that a 30% modifier was used. The time of extraction per sample was 45 min (step one 5 min, step two 40 min, and step three 10 min). The first step was performed to eliminate hydrocarbons and nonpolar compounds, the second step was performed to extract the insecticides, and the third step was performed to wash the thimble and ODS trap and to insure that no pesticides escaped in step two.

### Sample Analysis

All the pesticide residues were analyzed by Supercritical Fluid Chromatography (SFC/UVD) at 220 nm and extracted by SFE. The pesticide determination methods for pyrethroids[23,24], carbamates[23,25], and herbicides and fungicides[23,26] were modified [23] to meet the needs of the present investigation. Hence, a Hewlett Packard SFC model G 1205A attached to an HP 1050 diode array detector, modifier pump G 1205A and a silica column (Alltec Hypersil APS 25 micron, length 205 mm, ID 4.6 mm) was used. Pyrethroids were run at an oven temperature of 60 EC at a pressure of 130–200 bars (5 bar/min), flow rate was 1–3 ml/min at (2 ml/min), and 2% methanol was used as modifier peaks were detected at wavelength 220 nm. Herbicides and fungicides were run at an oven temperature of 30°C, at a pressure of 80–150 bars (30 bar/min), flow rate of 1–2 ml/min, 5 ml/min, and modifier 2–3% methanol (5%/min). Carbamates were run at an oven temperature of 32°C, at a pressure of 80 bars, flow rate of 1–2 ml/min (5 ml/min), and modifier 2–4% methanol (5%/min). Herbicides and carbamates were detected at 220 nm, while fungicides were detected at 210 nm. The overall methods were elaborated and verified by trials of several injections to obtain the best separation.

## RESULTS

### Pyrethroids

In 6 out of 15 analyzed samples, 4 different pyrethroid residues were detected, which ranged from 0.03 ± 0.005 to 0.05 ± 0.03 ppm. Most of these pyrethroid residues were detected in four samples of frozen vegetables and strawberry jam (Table 1A), with higher incidences of permethrin and deltamethrin in four different samples, but phenothrin and cypermethrin were detected in only two different samples. Allethrin, resmethrin, tetramethrin, and phenoxy benzyl alcohol are not detected in any food samples. Permethrin and deltamethrin residues were detected in strawberry jam (the same sample). In fact, nine samples namely: guava juice, mango juice, fruit cocktail juice, frozen artichoke, frozen aubergins, canned fava beans, fig and orange jams were free from any detected pyrethroid residues. Pyrethroid recovery and RSD percentages of spiked samples group ranged from 88.7 ± 2.8 to 99.6 ± 0.77 as found in Table 1B. The average of pyrethroid residue recovery ± RSD percentages ranged from 91.2 ± 1.5 to 99.3 ± 1.2 for juices, 93.6 ± 1.4 to 99.6 ± 0.77 for frozen vegetables, 92.2 ± 2.4 to 98.5 ± 1.6 for canned foods, and 88.7 ± 2.8 to 94.1 ± 1.3 for jams. The SFC run time was completely conducted in only 25 min.

**TABLE 1A**
**Pyrethroid Residues (ppm) and RSD in Food Samples Extracted by SFE and Determined by SFC/UVD at 220 nm**

| Food Samples | Pyrethroid Residues (ppm ± RSD) | | | |
|---|---|---|---|---|
| | **Allethrin** | **Resmethrin** | **Phenothrin** | **Permethrin** |
| Guava juice | ND | ND | ND | ND |
| Mango juice | ND | ND | ND | ND |
| Fruit cocktail juice | ND | ND | ND | ND |
| Frozen mixed veg. | ND | ND | ND | 0.04 ± 0.02 |
| Frozen molukhia | ND | ND | ND | ND |
| Frozen artichoke | ND | ND | ND | ND |
| Frozen colocassia | ND | ND | ND | ND |
| Frozen beans | ND | ND | ND | 0.03 ± 0.01 |
| Frozen aubergines | ND | ND | ND | ND |
| Frozen okra | ND | ND | 0.03 ± 0.005 | ND |
| Canned fava beans | ND | ND | ND | ND |
| Canned hot peppers | ND | ND | 0.04 ± 0.02 | ND |
| Fig jam | ND | ND | ND | ND |
| Orange jam | ND | ND | ND | ND |
| Strawberry jam | ND | ND | ND | 0.03 ± 0.02 |
| | **Tetramethrin** | **Cypermethrin** | **Deltamethrin** | **P.B.A** |
| Guava juice | ND | ND | ND | ND |
| Mango juice | ND | ND | ND | ND |
| Fruit cocktail juice | ND | ND | ND | ND |
| Frozen mixed veg. | ND | ND | ND | ND |
| Frozen molukhia | ND | ND | 0.05 ± 0.03 | ND |
| Frozen artichoke | ND | ND | ND | ND |
| Frozen colocassia | ND | ND | ND | ND |
| Frozen beans | ND | 0.03 ± 0.02 | ND | ND |
| Frozen aubergines | ND | ND | ND | ND |
| Frozen okra | ND | ND | ND | ND |
| Canned fava beans | ND | ND | ND | ND |
| Canned hot peppers | ND | ND | ND | ND |
| Fig jam | ND | ND | ND | ND |
| Orange jam | ND | ND | ND | ND |
| Strawberry jam | ND | ND | 0.05 ± 0.01 | ND |

ND: Not Detected.
P.B.A.: phenoxy benzyl alcohol

**TABLE 1B**
**Pyrethroid Recovery and RSD Percentages of Spiked Food Samples Extracted by SFE and Determined by SFC/UVD at 220 nm**

| Food Samples | Average Recovery and RSD% of Pyrethroids | | | |
|---|---|---|---|---|
| | **Allethrin** | **Resmethrin** | **Phenothrin** | **Permethrin** |
| Juices | 92.5 ± 1.8 | 94.7 ± 2.3 | 91.2 ± 1.5 | 96.3 ± 1.1 |
| Frozen vegetables | 96.7 ± 1.1 | 93.7 ± 1.1 | 93.6 ± 1.4 | 97.2 ± 2.1 |
| Canned foods | 96.2 ± 2.1 | 92.2 ± 2.4 | 92.7 ± 2.5 | 96.8 ± 1.7 |
| Jams | 89.4 ± 3.3 | 90.8 ± 2.6 | 89.5.9 ± 2.8 | 90.2 ± 2.4 |
| | **Tetramethrin** | **Cypermethrin** | **Deltamethrin** | **P.B.A.** |
| Juices | 93.3 ± 1.6 | 96.1 ± 1.4 | 99.3 ± 1.2 | 98.5 ± 0.86 |
| Frozen vegetables | 94.4 ± 1.4 | 97.8 ± 1.7 | 99.4 ± 1.4 | 99.6 ± 0.77 |
| Canned foods | 95.2 ± 2.4 | 96.9 ± 1.2 | 98.5 ± 1.6 | 98.3 ± 0.48 |
| Jams | 91.4 ± 2.3 | 88.7 ± 2.8 | 92.2 ± 2.2 | 94.1 ± 1.3 |

P.B.A.: phenoxy benzyl alcohol

### Herbicides

Herbicide residue results are shown in Table 2A. Herbicide residues in food samples ranged from 0.03 ± 0.005 to 0.8 ± 0.01 ppm. Eight different samples had four different herbicides namely: trifluralin, alachlor, atrazin, and simazin. Alachlor and atrazin were detected in four and five different samples, respectively. Trifluralin and simazin were detected in four and three different samples, respectively. However, four herbicides (tillam, chlorthal, propazin, and terbuthylazin) were not detected in the all the tested food samples. Furthermore, guava juice, frozen artichokes, frozen colocassia, canned fava beans, canned hot peppers, fig and orange jam samples were free from any detectable herbicides residues. The SFC run time was completely achieved in only 10 min. The recovery and RSD percentages of spiked samples in the herbicide group ranged from 87.7 ± 2.4 to 98.7 ± 1.6. The recovery ± RSD% ranged from 91.1 ± 1.5 to 98.3 ± 1.2 for juices, 93.6 ± 1.3 to 98.6 ± 1.7 for frozen vegetables, 92.7 ± 1.9 to 98.7 ± 1.6 for canned foods, and 88.9 ± 2.85 to 87.7 ± 2.4 for jams (Table 2B). The results showed no herbicide residues in 53.3% of the investigated food samples (Table 2A).

### Fungicides

Fungicide residue results are shown in Table 3A. Four fungicide residues detected in 30% of the investigated food samples, ranging from 0.05 ± 0.02 to 0.8 ± .01 ppm, were detected in five different frozen vegetable samples. PCNP and CDEC were detected only in three frozen vegetable samples. However, dichlon, captan, and thiram were not detected in all the investigated food samples. The SFC run time was completely achieved in only 12 min. The average recovery and RSD% of spiked samples in fungicide group ranged from 83.8 ± 2.1 to 98.6 ± 1.6. The fungicide residue recovery ± RSD% ranged from 84.3 ± 1.9 to 96.6 ± 1.5 for juices, 88.4 ± 1.4 to 98.6 ± 1.6 for frozen vegetables, 87.2 ± 1.5 to 98.7 ± 1.4 for canned foods, and 83.8 ± 2.1 to 90.5 ± 1.2 for jams (see Table 3B).

**TABLE 2A**
**Herbicide Residues (ppm) and RSD in Food Samples Extracted by SFE and Determined by SFC/UVD at 220 nm**

| Food Samples | Herbicide Residues (ppm ± RSD) | | | |
|---|---|---|---|---|
| | **Trifluralin** | **Tillam** | **Chlorthal** | **Alachlor** |
| Guava juice | ND | ND | ND | ND |
| Mango juice | ND | ND | ND | ND |
| Fruit cocktail juice | ND | ND | ND | ND |
| Frozen mixed veg. | 0.06 ± 0.01 | ND | ND | 0.04 ± 0.02 |
| Frozen molukhia | 0.4 ± 0.2 | ND | ND | 0.3 ± 0.05 |
| Frozen artichoke | ND | ND | ND | ND |
| Frozen colocassia | ND | ND | ND | ND |
| Frozen beans | 0.3 ± 0.1 | ND | ND | ND |
| Frozen aubergines | ND | ND | ND | 0.3 ± 0.01 |
| Frozen okra | ND | ND | ND | ND |
| Canned fava beans | ND | ND | ND | ND |
| Canned hot peppers | ND | ND | ND | ND |
| Fig jam | ND | ND | ND | ND |
| Orange jam | ND | ND | ND | ND |
| Strawberry jam | 0.6 ± 0.2 | ND | ND | 0.8 ± 0.01 |
| | **Propazin** | **Terbuthylazin** | **Atrazin** | **Simazin** |
| Guava juice | ND | ND | ND | ND |
| Mango juice | ND | ND | 0.03 ± 0.005 | 0.04 ± 0.02 |
| Fruit cocktail juice | ND | ND | ND | ND |
| Frozen mixed veg. | ND | ND | 0.07 ± 0.01 | ND |
| Frozen molukhia | ND | ND | 0.06 ± 0.01 | ND |
| Frozen artichoke | ND | ND | ND | ND |
| Frozen colocassia | ND | ND | ND | ND |
| Frozen beans | ND | ND | 0.6 ± 0.02 | ND |
| Frozen aubergines | ND | ND | ND | ND |
| Frozen okra | ND | ND | ND | 0.09 ± 0.01 |
| Canned fava beans | ND | ND | ND | ND |
| Canned hot peppers | ND | ND | ND | ND |
| Fig jam | ND | ND | ND | ND |
| Orange jam | ND | ND | ND | ND |
| Strawberry jam | ND | ND | 0.05 ± 0.03 | 0.4 ± 0.2 |

ND: Not Detected.

**TABLE 2B**
**Herbicide Recovery and RSD Percentages of Spiked Food Samples Extracted by SFE and Determined by SFC/UVD at 220 nm**

| Food Samples | Average Recovery and RSD% of Herbicides | | | |
|---|---|---|---|---|
| | **Trifluralin** | **Tillam** | **Chlorthal** | **Alachlor** |
| Juices | 96.1 ± 1.2 | 91.2 ± 1.7 | 95.4 ± 2.3 | 96.5 ± 1.8 |
| Frozen vegetables | 96.8 ± 1.4 | 93.6 ± 1.3 | 97.6 ± 1.1 | 98.6 ± 1.7 |
| Canned foods | 95.7 ± 1.6 | 92.7 ± 1.9 | 97.8 ± 2.4 | 98.3 ± 1.4 |
| Jams | 87.7 ± 2.4 | 88.9 ± 2.5 | 91.1 ± 2.7 | 90.1 ± 1.7 |
| | **Propazin** | **Terbuthylazin** | **Atrazin** | **Simazin** |
| Juices | 94.5 ± 1.4 | 91.1 ± 1.5 | 92.3 ± 1.6 | 98.3 ± 1.2 |
| Frozen vegetables | 95.7 ± 1.9 | 94.2 ± 1.3 | 95.4 ± 1.6 | 97.1 ± 1.4 |
| Canned foods | 95.2 ± 2.2 | 93.8 ± 1.7 | 97.2 ± 1.3 | 98.7 ± 1.6 |
| Jams | 90.4 ± 2.2 | 92.2 ± 1.8 | 89.8 ± 2.7 | 91.7 ± 2.4 |

**TABLE 3A**
**Fungicide Residues (ppm) and RSD in Food Samples Extracted by SFE and Determined by SFC/UVD at 210 nm**

| Food Samples | Fungicide Residues (ppm ± RSD) | | | | | | |
|---|---|---|---|---|---|---|---|
| | **PCNP** | **CDEC** | **Dichlon** | **Captan** | **Captafol** | **Thiram** | **Carboxin** |
| Guava juice | ND | ND | ND | ND | ND | ND | ND |
| Mango juice | ND | ND | ND | ND | ND | ND | ND |
| Fruit cocktail juice | ND | ND | ND | ND | ND | ND | ND |
| Frozen mixed veg. | 0.05 ± 0.02 | ND | ND | ND | ND | ND | ND |
| Frozen molukhia | ND | ND | ND | ND | ND | ND | 0.5 ± 0.2 |
| Frozen artichoke | ND | ND | ND | ND | 0.3 ± 0.05 | ND | ND |
| Frozen colocassia | ND | ND | ND | ND | ND | ND | ND |
| Frozen beans | 0.7 ± 0.1 | 0.4 ± 0.2 | ND | ND | ND | ND | 0.2 ± 0.05 |
| Frozen aubergines | ND | ND | ND | ND | 0.3 ± 0.1 | ND | ND |
| Frozen okra | 0.7 ± 0.1 | 0.8 ± 0.1 | ND | ND | 0.6 ± 0.1 | ND | 0.7 ± 0.1 |
| Canned fava beans | ND | ND | ND | ND | ND | ND | ND |
| Canned hot peppers | ND | ND | ND | ND | ND | ND | ND |
| Fig jam | ND | ND | ND | ND | ND | ND | ND |
| Orange jam | ND | ND | ND | ND | ND | ND | ND |
| Strawberry jam | ND | ND | ND | ND | ND | ND | ND |

ND: Not Detected.

**TABLE 3B**
**Fungicide Recovery and RSD Percentages of Spiked Food Samples Extracted by SFE and Determined by SFC/UVD at 210 nm**

| Food Samples | Average Recovery and RSD% of Fungicides | | | | | | |
|---|---|---|---|---|---|---|---|
| | PCNP | CDEC | Dichlon | Captan | Captafol | Thiram | Carboxin |
| Juices | 87.3 ± 1.3 | 96.1 ± 1.4 | 89.3 ± 1.6 | 96.6 ± 1.5 | 98.2 ± 1.7 | 90.4 ± 1.8 | 84.3 ± 1.9 |
| Frozen vegetables | 89.7 ± 1.4 | 96.3 ± 1.7 | 90.4 ± 1.6 | 95.8 ± 1.7 | 98.6 ± 1.6 | 91.6 ± 1.3 | 88.4 ± 1.4 |
| Canned foods | 89.2 ± 2.8 | 97.4 ± 1.6 | 91.2 ± 1.3 | 94.7 ± 1.3 | 98.7 ± 1.4 | 92.8 ± 1.5 | 87.2 ± 1.5 |
| Jams | 86.1 ± 2.4 | 90.5 ± 1.2 | 85.4 ± 2.1 | 86.7 ± 2.8 | 89.5 ± 2.7 | 90.1 ± 2.4 | 83.8 ± 2.1 |

### Carbamates

Carbamate residue results are shown in Table 4A. Of the food samples under this investigation, 60% had no detection of carbamate residues. Six different samples had six different carbamate residues ranging from 0.04 ± 0.02 to 0.8 ± 0.1 ppm. However, all the investigated carbamates were not detected in three frozen vegetables, two canned foods, and three jam samples. The presence of oxamyl was detected in six different samples, benthiocarb and aldicarb were detected in three samples, but barban was only detected in one sample. The average recovery and RSD% of spiked samples by 12 carbamate groups ranged from 80.2 ± 3.8 to 98.8 ± 1.7. The individual carbamate recovery ± RSD% ranged from 80.3 ± 1.8 to 98.8 ± 1.7 for juices, 84.2 ± 2.3 to 98.6 ± 1.3 for frozen vegetables, 83.8 ± 2.7 to 98.7 ± 1.5 for canned foods, and 80.2 ± 3.8 to 92.1 ± 2.2 for jams. The SFC run time was completely achieved in only 30 min.

### DISCUSSION

The levels of pesticide residues in canned foods, vegetables, and fruits have been determined using modified SFE and SFC techniques. Groups (4) of pesticides (8 pyrethroids and their metabolites, 8 herbicides, 7 fungicides, and 12 carbamates) that were used in agriculture were studied. The extraction method by SFE was fast, safe, high-recovery percentage, and inexpensive for all four groups of pesticide residues. We were able to obtain the optimum conditions that enabled us to extract a total of 35 pesticides in each freeze-dried food sample (control or spiked) in 55 min. The determinations were fast, no clean-up exercise was needed, and lowest detection limit (ppb) was achieved for each pesticide residue (Tables 1A, 2A, 3A, and 4A). The extraction method by SFE was fast, safe, inexpensive, and the percentage of recovery was high.

The application of SFC techniques in the analysis of pesticide residues in canned foods, fruits, and vegetables has been investigated. Pyrethroid recovery and RSD percentages of spiked sample groups ranged from 88.7 ± 2.8 to 99.6 ± 0.77 as found in Table 1B. The average of pyrethroid residue recovery ± RSD percentages ranged from 91.2 ± 1.5 to 99.3 ± 1.2 for juices, 93.6 ± 1.4 to 99.6 ± 0.77 for frozen vegetables, 92.2 ± 2.4 to 98.5 ± 1.6 for canned foods, and 88.7 ± 2.8 to 94.1 ± 1.3 for jams. Herbicide residues in food samples ranged from 0.03 ± 0.005 to 0.8 ± 0.01 ppm. The recovery and RSD percentages of spiked samples in the herbicide group ranged from 87.7 ± 2.4 to 98.7 ± 1.6. The results showed no herbicide residues in 53.3% of the investigated food samples. Fungicide recoveries ranged from 84.3 ± 1.9 to 96.6 ± 1.5 for juices, 88.4 ± 1.4 to 98.6 ± 1.6 for

**TABLE 4A**
**Carbamate Residues (ppm) and RSD in Food Samples Extracted by SFE and Determined by SFC/UVD at 220 nm**

| Food Samples | Carbamate Residues (ppm ± RSD) | | | | | |
|---|---|---|---|---|---|---|
| | **Benthiocarb** | **Pirimicarb** | **Aldicarb** | **Bendiocarb** | **Baygon** | **BDMC** |
| Guava juice | ND | ND | 0.04 ± 0.02 | ND | ND | ND |
| Mango juice | ND | ND | ND | ND | ND | ND |
| Fruit cocktail juice | ND | ND | 0.3 ± 0.1 | ND | ND | ND |
| Frozen mixed veg. | ND | ND | ND | ND | ND | ND |
| Frozen molukhia | ND | ND | 0.8 ± 0.1 | ND | 0.2 ± 0.05 | ND |
| Frozen artichoke | ND | ND | ND | ND | ND | ND |
| Frozen colocassia | ND | ND | ND | ND | ND | ND |
| Frozen beans | 0.2 ± 0.005 | ND | ND | ND | ND | ND |
| Frozen aubergines | 0.1 ± 0.03 | ND | ND | ND | ND | ND |
| Frozen okra | 0.8 ± 0.1 | ND | ND | ND | 0.5 ± 0.3 | ND |
| Canned fava beans | ND | ND | ND | ND | ND | ND |
| Canned hot peppers | ND | ND | ND | ND | ND | ND |
| Fig jam | ND | ND | ND | ND | ND | ND |
| Orange jam | ND | ND | ND | ND | ND | ND |
| Strawberry jam | ND | ND | ND | ND | ND | ND |
| | **Methiocarb** | **Barban** | **Carbaryl** | **Benomyl** | **Methomyl** | **Oxamyl** |
| Guava juice | ND | ND | ND | ND | ND | 0.4±0.2 |
| Mango juice | ND | ND | ND | ND | ND | ND |
| Fruit cocktail juice | ND | ND | 0.5 ± 0.2 | ND | ND | ND |
| Frozen mixed veg. | ND | ND | ND | ND | ND | ND |
| Frozen molukhia | ND | ND | 0.3 ± 0.1 | ND | ND | ND |
| Frozen artichoke | ND | ND | ND | ND | ND | ND |
| Frozen colocassia | ND | ND | ND | ND | ND | ND |
| Frozen beans | ND | 0.4 ± 0.2 | 0.8 ± 0.1 | ND | ND | ND |
| Frozen aubergines | ND | ND | ND | ND | ND | 0.4 ± 0.2 |
| Frozen okra | ND | ND | ND | ND | ND | 0.7 ± 0.2 |
| Canned fava beans | ND | ND | ND | ND | ND | ND |
| Canned hot peppers | ND | ND | ND | ND | ND | ND |
| Fig jam | ND | ND | ND | ND | ND | ND |
| Orange jam | ND | ND | ND | ND | ND | ND |
| Strawberry jam | ND | ND | ND | ND | ND | ND |

ND: Not Detected.

frozen vegetables, 87.2 ± 1.5 to 98.7 ± 1.4 for canned foods, and 83.8 ± 2.1 to 90.5 ± 1.2 for jams, compared to other techniques[30,31,32,33] with a recovery range from 52.5 to 91.1% with RSDs between 6.1 and 11.9%. The average recovery and RSD% of spiked samples by 12 carbamate groups ranged from 80.2 ± 3.8 to 98.8 ± 1.7. The overall SFC run time was completely achieved within 10–30 min.

Monitoring pesticide residues in foods, fruits, and vegetables helps to assess the potential risk of these products to consumer health and gives information on the pesticide treatments that have been used on the processes of agricultural harvesting, preservation, and distribution[27]. Although other methods have been used to determine fungicides in fruits, vegetables, and canned foods[28], our techniques are more precise, cost effective, and less time consuming compared to others[29,30,31].

### CONCLUSION

Fifteen different types of canned and frozen fruits and vegetables samples obtained from the Houston local food markets were investigated. A fast and reliable method for the determination of more than 60% these food samples showed no detection of any investigated pesticide residues. On the other hand, 39.15% of the investigated foods found four pyrethroids, four herbicides, four fungicides, and six carbamates in different food samples with different levels of residues using the SFE for the extraction and SFC for the determination. The continuous sequential decolorization of the organic phase, solvent changeover, and solid-phase extraction for clean up were not required.

Trace levels of 35 pesticides in the investigated blank and spiked food samples can be extracted in only 55 min by our modified multiresidues SFE method. Determination method by SFC is considerably fast with low detection limit. The SFC run time for the 4 pesticide groups (8 pyrethroids, 8 herbicides, 7 fungicides, and 12 carbamates) was completely achieved in 25, 10, 12, and 30 min, respectively. The residue results indicated that, in general, the juices, frozen vegetables, canned foods, and jam samples had different levels of pesticide residues ranging from 0.03 ± 0.005 to 0.8 ± 0.12 ppm. However, high recovery percentage was also achieved. These results demonstrated the usefulness of the SFE and SFC multi methods for extraction and determination of four different groups of pesticide residues in a short time, LDL, high recovery and environmentally safer in all investigated food samples. Also to contribute to the promotion of consumer safety by excluding pesticide residues contamination from markets.

**TABLE 4B**
**Carbamate Recovery and RSD Percentages of Spiked Food Samples Extracted by SFE and Determined by SFC/UVD at 220 nm**

| Food Samples | Average Recovery and RSD% of Carbamates | | | | | |
|---|---|---|---|---|---|---|
| | **Benthiocarb** | **Pirimicarb** | **Aldicarb** | **Bendiocarb** | **Baygon** | **BDMC** |
| Juices | 97.6 ± 1.7 | 98.8 ± 1.7 | 94.4 ± 1.2 | 94.3 ± 1.5 | 95.1 ± 1.2 | 96.2 ± 1.4 |
| Frozen vegetables | 96.8 ± 1.4 | 98.3 ± 1.2 | 95.8 ± 1.7 | 98.4 ± 1.7 | 96.5 ± 1.4 | 98.6 ± 1.3 |
| Canned foods | 96.3 ± 1.4 | 98.4 ± 1.2 | 95.4 ± 1.3 | 97.2 ± 1.2 | 95.8 ± 1.3 | 98.7 ± 1.5 |
| Jams | 92.1 ± 2.2 | 89.6 ± 1.8 | 90.5 ± 2.7 | 88.8 ± 2.8 | 87.4 ± 2.7 | 89.9 ± 2.4 |
| | **Methiocarb** | **Barban** | **Carbaryl** | **Benomyl** | **Methomyl** | **Oxamyl** |
| Juices | 94.4 ± 2.3 | 93.5 ± 1.7 | 90.5 ± 1.2 | 81.1 ± 2.5 | 80.3 ± 1.8 | 96.3 ± 1.6 |
| Frozen vegetables | 96.6 ± 1.1 | 95.6 ± 1.4 | 93.5 ± 1.6 | 84.2 ± 2.3 | 85.4 ± 1.6 | 97.1 ± 1.3 |
| Canned foods | 95.8 ± 2.4 | 95.3 ± 1.8 | 92.2 ± 1.2 | 83.8 ± 2.7 | 87.2 ± 2.3 | 98.7 ± 1.8 |
| Jams | 90.1 ± 3.2 | 90.5 ± 1.6 | 89.4 ± 2.6 | 80.2 ± 3.8 | 85.8 ± 2.9 | 9 0.7 ± 2.9 |

## References

1. Akiyama, Y., Yoshioka, N., and Tsuji, M. (2002) Pesticide residues in agricultural products monitored in Hyogo Prefecture, Japan, FYs 1995–1999. *J. AOAC Int.* **85(3),** 692–703.
2. Yen, I.C., Bekele, I., and Kalloo, C. (1999) Use patterns and residual levels of organophosphate pesticides on vegetables in Trinidad, West Indies. *J. AOAC Int.* **82(4),** 991–995.
3. Neidert, E. and Saschenbrecker, P.W. (1996) Occurrence of pesticide residues in selected agricultural food commodities available in Canada. *J. AOAC Int.* **79(2),** 549–566.
4. Ekstrom, G., Hemming, H., and Palmborg, M. (1996) Swedish pesticide risk reduction 1981–1995: food residues, health hazard, and reported poisonings. *Rev. Environ. Contam. Toxicol.* **147,** 119–147.
5. Roy, R.R., Wilson, P., Laski, R.R., Roberts, J.I., Weishaar, J.A., Bong, R.L., and Yess, N.J. (1997) Monitoring of domestic and imported apples and rice by the U.S. Food and Drug Administration pesticide program. *J. AOAC Int.* **80(4),** 883–894.
6. Roy, R.R., Albert, R.H., Wilson, P., Laski, R.R., Roberts, J.I., Hoffmann, T.J., Bong, R.L., Bohannon, B.O., and Yess, N.J. (1995) U.S. Food and Drug Administration pesticide program: incidence/level monitoring of domestic and imported pears and tomatoes. *J. AOAC Int.* **78(4),** 930–940.
7. EL-Saeid, M.H. and Shaht, M. (2000) Detection of pesticide residues and heavy metals in some fresh fruits and vegetables collected from Cairo. 1st Mansoura Conf. of Food and Dairy Tech., 17–19 October, Cairo, Egypt. pp. 183–203.
8. Torres, C.M., Pico, Y., and Manes, J. (1996) Determination of pesticide residues in fruit and vegetables. *J.Chromatogr. A* **754(1–2),** 301–331.
9. Albero, B., Brunte, C.S., and Tadeo, J.L. (2001) Multiresidue determination of pesticides in honey by matrix solid-phase dispersion and gas chromatography with electron-capture detection. *J. AOAC Int.* **84(4),** 1165–1171.
10. Tadeo, J.L., Sanchez-Brunete, C., Perez, R.A., and Fernandez, M.D. (2000) Analysis of herbicide residues in cereals, fruits and vegetables. *J. Chromatogr. A* **882(1–2),** 175–191.
11. Colume, A., Cardenas, S., Gallego, M., and Valcarcel, M. (2000) Simplified method for the determination of chlorinated fungicides and insecticides in fruits by gas chromatography. *J. Chromatogr. A* **882(1–2),** 193–203.
12. Navarro, S., Barba, A., Navarro, G., Vela, N., and Oliva, J. (2000) Multiresidue method for the rapid determination—in grape, must and wine—of fungicides frequently used on vineyards. *J. Chromatogr. A* **882(1–2),** 221–229.
13. Fernandez, M., Pico, Y., and Manes, J. (2000) Determination of carbamate residues in fruits and vegetables by matrix solid-phase dispersion and liquid chromatography-mass spectrometry. *J. Chromatogr. A* **871(1–2),** 43–56.
14. Ripley, B.D., Ritcey, G.M., Harris, C.R., Denomme, M.A., and Lissemore, L.I. (2003) Comparative persistence of pesticides on selected cultivars of specialty vegetables. *J. Agric. Food Chem.* **51(5),** 1328–1335.
15. Valenzuela, A.I., Pico, Y., and Font, G. (2001) Determination of five pesticide residues in oranges by matrix solid-phase dispersion and liquid chromatography to estimate daily intake of consumers. *J. AOAC Int.* **84(3),** 901–909.
16. Horwitz, W., Jackson, T., and Chirtel, S.J. (2001) Examination of proficiency and control recovery data from analyses for pesticide residues in food: sources of variability. *J. AOAC Int.* **84(3),** 919–935.
17. Chen, Z.M. and Wang, Y.H. (1996) Chromatographic methods for the determination of pyrethrin and pyrethroid pesticide residues in crops, foods and environmental samples. *J. Chromatogr. A* **754,** 367–395.
18. Ahmed, M.T., Ismail, S.M., and Mosleh, Y.Y. (1998) Determination of malathion residues in some medicinal plants by liquid chromatography with gas chromatographic/mass spectrometric confirmation. *J. AOAC Int.* **81(5),** 1023–1026.
19. Aharonson, N. and Kafkafi, U. (1975) Adsorption of benzimidazole fungicides on montmorillonite and kaolinite clay surfaces. *J. Agric. Food Chem.* **23(3),** 434–437.
20. Nelson, J. and Abdelmessah, H. (1992). Laboratory Information Bulletin, FDA, Brooklyn, NY, 11232.
21. Khan, S.U. (1982) Bound pesticide residues in soil and plants. *Residue Rev.* **84,** 1–25.
22. Ashraf, S., Bartle, K.D., Clifford, A.A., and Moulder, R. (1991) Trace analysis of agrochemicals by supercritical fluid chromatography. *J. High Resol. Chromatogr.* **14,** 29–32.
23. EL-Saeid, M.H. (1999) New Techniques for Residue Analysis of Pesticides in Foods [Ph.D. Dissertation]. Al-Azhar University, Cairo, Egypt.
24. Nishikawa, Y. (1993). Retention behavior of synthetic pyrethroids in capillary supercritical fluid chromatography. *Anal. Sci.* **9**, 39–42.
25. Beverly, M.B., Basile, F., Voorhees, K.J., and Hadfield, T.L. (1996) A rapid approach for the detection of dipicolinic acid in bacterial spores using pyrolysis/mass spectrometry. *Rapid Commun. Mass Spectrom.* **10(4),** 455–458.

26. France, J.E. and Voorhees, K.J. (1988) Capillary supercritical fluid chromatography with ultraviolet multichannel detection of some pesticides and herbicides. *J. High Resol. Chromatogr. Chromatogr. Commun.* **11,** 692–696.
27. Fernandez, M., Rodriguez, R., Pico, Y., and Manes, J. (2001) Liquid chromatographic-mass spectrometric determination of post-harvest fungicides in citrus fruits. *J. Chromatogr. A* **912(2),** 301–310.
28. FDA (1993) Monitoring Program. *J. AOAC Int.* **76(5),** 127A–148A.
29. Pico, Y., Font, G., Molto, J.C., and Manes, J. (2000) Pesticide residue determination in fruit and vegetables by liquid chromatography-mass spectrometry. *J. Chromatogr. A* **882(1–2),** 153–173.
30. Valenzuela, A.I., Pico, Y., and Font, G. (2000) Liquid chromatography/atmospheric pressure chemical ionization-mass spectrometric analysis of benzoylurea insecticides in citrus fruits. *Rapid Commun. Mass. Spectrom*. **14(7),** 572–577.
31. Blasco, C., Pico, Y., and Font, G. (2002) Monitoring of five postharvest fungicides in fruit and vegetables by matrix solid-phase dispersion and liquid chromatography/mass spectrometry. *J. AOAC Int.* **85(3),** 704–711.
32. Blanco, C., Pico, Y., Manes, J., and Font, G. (2002) Determination of fungicide residues in fruits and vegetables by liquid chromatography-atmospheric pressure chemical ionization mass spectrometry. *J. Chromatogr. A* **947(2),** 227–235.
33. Valenzuela, A.I., Redondo, M.J., Pico, Y., and Font, G. (2000) Determination of abamectin in citrus fruits by liquid chromatography-electrospray ionization mass spectrometry. *J. Chromatogr. A* **871(1–2),** 57–65.

### BIOSKETCH

Mohamed El-Saeid is a Visiting Professor/Research Associate and a member of the Biomonitor Research Group, Department of Chemistry, Texas Southern University, Houston, Texas. Dr. El-Saeid was a Post Doctoral and Research Associate, Department of Chemistry, Oklahoma State University and also was Assistant Professor in Food Sanitation and Pesticide Chemistry in the Department of Food Science and Technology, Faculty of Agriculture at Al-Azhar University in Egypt.
